# Bone mineral density and cardiovascular diseases: a two-sample Mendelian randomization study

**DOI:** 10.1093/jbmrpl/ziaf037

**Published:** 2025-03-03

**Authors:** Ahmed M Salih, Dorina-Gabriela Condurache, Stefania D’Angelo, Elizabeth M Curtis, Steffen E Petersen, Andre Altmann, Nicholas C Harvey, Zahra Raisi-Estabragh

**Affiliations:** William Harvey Research Institute, NIHR Barts Biomedical Research Centre, Queen Mary University of London, London EC1M 6BQ, United Kingdom; Department of Population Health Sciences, University of Leicester, Leicester LE1 7RH, United Kingdom; PRIME Lab, Scientific Research Center, University of Zakho, Zakho, Kurdistan Region, Iraq; Barts Heart Centre, St Bartholomew’s Hospital, Barts Health NHS Trust, London EC1A 7BE, United Kingdom; William Harvey Research Institute, NIHR Barts Biomedical Research Centre, Queen Mary University of London, London EC1M 6BQ, United Kingdom; Barts Heart Centre, St Bartholomew’s Hospital, Barts Health NHS Trust, London EC1A 7BE, United Kingdom; MRC Lifecourse Epidemiology Centre, University of Southampton, Southampton SO16 6YD, United Kingdom; MRC Lifecourse Epidemiology Centre, University of Southampton, Southampton SO16 6YD, United Kingdom; NIHR Southampton Biomedical Research Centre, University of Southampton and University Hospital Southampton NHS Foundation Trust, Southampton SO16 6YD, United Kingdom; William Harvey Research Institute, NIHR Barts Biomedical Research Centre, Queen Mary University of London, London EC1M 6BQ, United Kingdom; Barts Heart Centre, St Bartholomew’s Hospital, Barts Health NHS Trust, London EC1A 7BE, United Kingdom; Department of Medical Physics and Biomedical Engineering, The UCL Hawkes Institute, University College London, London WC1E 6BT, United Kingdom; MRC Lifecourse Epidemiology Centre, University of Southampton, Southampton SO16 6YD, United Kingdom; NIHR Southampton Biomedical Research Centre, University of Southampton and University Hospital Southampton NHS Foundation Trust, Southampton SO16 6YD, United Kingdom; William Harvey Research Institute, NIHR Barts Biomedical Research Centre, Queen Mary University of London, London EC1M 6BQ, United Kingdom; Barts Heart Centre, St Bartholomew’s Hospital, Barts Health NHS Trust, London EC1A 7BE, United Kingdom

**Keywords:** cardiovascular diseases, osteoporosis, Mendelian randomization, genome-wide association study, BMD, causality

## Abstract

The link between BMD and cardiovascular disease (CVD) remains a topic of extensive debate in observational studies, with inconsistent reports regarding the causality of this relationship. This study implements robust methodologies to evaluate the causal relationship between BMD and various CVDs. Two sample Mendelian randomization (MR) method was used to estimate the relationship between genetically predicted BMD and seven key CVDs: atrial fibrillation and flutter, angina, ischemic heart disease, heart failure, hypertension, myocardial infarction, and non-ischemic cardiomyopathy. Data were obtained from independent publicly available genome-wide association studies (GWAS) for BMD and CVDs, using two separate datasets for the cardiovascular outcomes: the UK Biobank cohort (primary analysis) and the FinnGen cohort (validation analysis). The MR Pleiotropy RESidual Sum and Outlier test assessed the heterogeneity and pleiotropy of selected instrumental variables (IVs). We applied the inverse variance weighted model (IVW), weighted median, weighted mode method, and MR-Egger regression model to estimate causal effects. MR results indicate no relationship between BMD and atrial fibrillation and flutter (IVW, beta-estimate: 0.011, SE: 0.03, *p* = .73), angina (IVW, beta-estimate: 0.04, SE: 0.03, *p* = .17), chronic ischemic heart disease (IVW, beta-estimate: 0.009, SE: 0.03, *p* = .74), heart failure (IVW, beta-estimate: 0.004, SE: 0.04, *p* = .91), hypertension (IVW, beta-estimate: −0.01, SE: 0.01, *p* = .44), myocardial infarction (IVW, beta-estimate: 0.02, SE: 0.03, *p* = .36), or non-ischemic cardiomyopathy (IVW, beta-estimate: 0.1, SE: 0.08, *p* = .20). These findings remained consistent across all complementary analyses (MR-Egger, weighted median and weighted mode) and were validated using the FinnGen cohort GWAS dataset. This comprehensive analysis identified no evidence for a causal link between genetically predicted BMD and a range of key CVDs. Previously reported observational associations between bone and cardiovascular health likely represent shared risk factors rather than direct causal mechanisms.

## Graphical Abstract

**Graphical Abstract:**
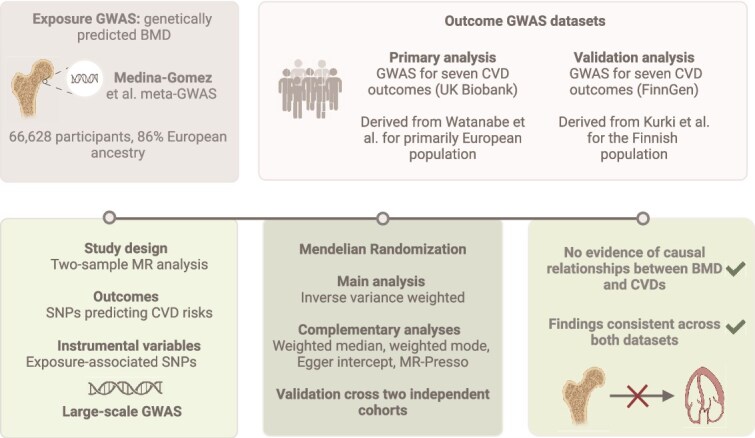

## Introduction

Cardiovascular disease (CVD) remains the leading cause of mortality and morbidity worldwide, responsible for 20.5 million deaths in 2021.^1^ As the global population continues to age, the burden of CVD is expected to increase, leading to substantial public health and societal challenges.^2^ Similarly, osteoporosis is the most common chronic metabolic disorder characterized by reduced bone mass and a heightened risk of fragility fractures.^3^ It is a significant health issue, particularly among older adults and postmenopausal women, due to its impact on quality of life, healthcare costs and the risk of debilitating fractures. Currently, osteoporosis affects approximately 500 million people worldwide and this number is expected to rise in the coming decades, driven by aging populations and longer life expectancies.^4^^,^^5^

Traditionally, osteoporosis and CVD were viewed as separate conditions with distinct age-related pathophysiological processes. However, increasing evidence suggests a potential link between the two beyond age and shared risk factors. Several observational studies have identified associations between BMD and CVD occurrence. For instance, Fohtung et al.^6^ found that lower total hip BMD was linked to an increased risk of heart failure. Other studies have reported an association between reduced BMD and greater incidence of coronary heart disease (CHD),^7^ acute myocardial infarction,^8^ mortality and stroke.^9^ However, it can be difficult to ascertain the causal nature of these relationships due to potential biases inherent in observational data. Mendelian randomization (MR) offers a unique opportunity to overcome these limitations using genetic variants as instrumental variables (IV).^10^

MR uses the random assortment of genetic material at conception to create a natural experiment, which eliminates the influence of confounding and reverse causation.^10^ Using this technique, we can group people according to their genetic code and more confidently assert relationships of interest. Instead of measuring expressed phenotypes, this technique tests relationships between genetically predicted characteristics defined using suitable IVs. For IVs to be valid in MR analysis, they should meet specific criteria: they should be independent of any confounding (observed or unobserved) variables, significantly associated with the exposure, and be associated with the outcome only through the exposure.^11^ In recent years, two-sample MR has generally been established as best practice. This design uses genetic IVs for the exposure and outcome derived from two independent non-overlapping samples and has the key advantage of increasing statistical power. Other MR methods have also emerged, which act as sensitivity checks for the main primary analysis.

Several MR studies have assessed the relationship between BMD and individual CVD outcomes, such as coronary artery calcification,^12^ CHD,^13^^,^^14^ myocardial infarction, stroke^13^ and heart failure.^15^ Whilst one study, which used ultrasound-based estimated BMD, reported a causal effect of BMD on CHD,^14^ studies employing DXA-derived BMD phenotypes have found no causal relationship between BMD and coronary artery calcification,^12^ CHD, MI, stroke^13^ and heart failure.^15^ Interestingly, bidirectional MR analyses revealed a causal effect in the reverse direction, where CHD and HF were shown to influence BMD.^13^^,^^14^

Our study aims to clarify the causal relationship between genetically predicted BMD and a comprehensive range of CVDs using a two-sample MR approach. By incorporating dual GWAS datasets for CVD outcomes from the UK Biobank (primary analysis) and FinnGen (validation analysis), we aim to provide a more nuanced understanding of the skeletal-cardiovascular interplay, addressing the limitations and methodological variations observed in previous studies.

The main objective of this study is to explore genetic associations of BMD and seven key CVDs, including atrial fibrillation (AF) and flutter, angina, chronic ischemic heart disease (IHD), heart failure, hypertension, myocardial infarction and non-ischemic cardiomyopathy (NICM), using a two-sample MR framework. This study represents the first comprehensive MR analysis of the causal links between BMD and CVDs, providing valuable insights that could inform clinical practice and preventive strategies.

## Materials and methods

### Study design

**Figure 1:**
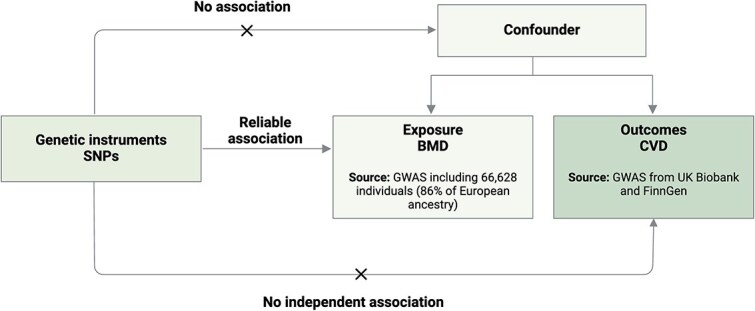
Overall study design. Schematic representation of the MR analysis performed SNPs: single nucleotide polymorphisms. Abbreviations: GWAS: genome-wide association study; IVW: inverse variance weighted; LD: linkage disequilibrium; MR: Mendelian randomization; SE: standard error.

In this genetic association study, we implemented a two-sample MR approach that uses genetic variants as IVs for the exposure to explore whether BMD may be causally associated with CVD outcomes (Figure 1). We ensured that the populations in the exposure and outcome genome-wide association study (GWAS) were comparable and that there was no overlap between cohorts, minimizing potential biases. All GWAS data in this study were publicly available, with the informed consent and ethical approval previously obtained. This MR study followed the Strengthening the Reporting of Observational Studies in Epidemiology using Mendelian Randomization (STROBE-MR) guidelines.^16^

### Data sources

#### GWAS summary statistics for BMD (exposure)

We used summary statistics from a GWAS meta-analysis for total body BMD (TB-BMD) conducted by Medina-Gomez et al.,^17^ which included data from 66 628 individuals across 30 cohorts in Europe, Australia, and America. The majority (86%) of participants were of European ancestry, providing a robust dataset for genetic analysis. We employed only the summary statistics from the European cohort to minimize potential biases arising from population structure. Although there is a preference in clinical settings for measuring BMD at specific sites, TB-BMD assesses BMD across the entire skeleton, allowing for the capture of a wider range of genetic influences compared to localized measurement. Additionally, the GWAS dataset for TB-BMD is one of the largest available, and the substantial sample size improves the statistical power of the study, enabling the detection of genetic associations with increased reliability.

#### GWAS summary statistics for CVD (outcomes)

All CVD datasets were derived from the UK Biobank, with a considerable number of cases and controls for each condition, ensuring robust statistical power. For angina (12 114 cases and 373 585 controls), hypertension (99 665 cases and 189 642 controls), chronic IHD (14 456 cases and 286 335 controls) and AF and flutter (10 986 cases and 233 904 controls), we referred to data obtained from Watanabe et al.^18^ Data for heart failure (6504 cases and 387 652 controls) and NICM (1816 cases and 388 326 controls) were obtained from Aragam et al.^19^ Lastly, myocardial infarction data (17 505 cases and 454 212 controls) were obtained from Hartiala et al.^20^ The GWAS summary statistics for all cardiac conditions were downloaded from GWAS Atlas,^18^ apart from myocardial infarction, which was downloaded from GWAS Catalog.^21^

To validate our findings, we used the FinnGen GWAS database, which included 224 737 participants of Finnish ancestry (https://www.finngen.fi/en). The FinnGen consortium is an ongoing project launched in Finland in 2017, collecting genetic and electronic health record information, aiming to explore the human genome.^22^ Seven CVDs were used as outcomes in this study. These included angina (36 875 cases and 343 079 controls), hypertension (122 996 cases and 289 117 controls), chronic IHD (69 008 cases and 343 173 controls), AF and flutter (50 743 cases and 210 652 controls), heart failure (29 218 cases and 381 838 controls), NICM (10 839 cases and 332 334 controls), and myocardial infarction (26 060 cases and 343 079 controls). A detailed description of the studies included and the corresponding ICD10 codes used to define the CVD outcomes is presented in Tables S1–S3.

### Selection of instrumental variables

In our MR analysis, single nucleotide polymorphisms (SNPs) served as instrumental variables (IVs). A screening protocol for IVs was applied, including (1) extraction of genetic variants that met the GWAS *p*-value threshold for genome-wide significance (*p* < 5 × 10^−8^) and (2) application of linkage disequilibrium (LD) clumping with an *r*^2^ = 0.001 and a 10 000 kb windows size to guarantee the independence of these variants. Moreover, we obtained GWAS summary data for cardiac conditions using the same group of SNPs. By ensuring the robustness of IVs through these stringent selection criteria, we aimed to enhance the reliability and validity of our MR analysis.

### Power calculations

We conducted a two-sample MR to evaluate the causal association between BMD (Non-UK Biobank) as the exposure and the seven cardiac conditions as outcomes (using data from UK Biobank and FinnGen). Power calculations were conducted to determine whether our study was adequately powered to detect true causal effects between BMD and CVD outcomes (Table S4). The power of the MR estimates was calculated using the online tool mRnd: Power calculations for MR (shiny.cnsgenomics.com/mRnd/) to ascertain the power of finding MR connections.^23^ This approach considers the number of cases in the binary outcome GWAS and the variance in the exposure attributed to the instrumental variables. The statistical power was calculated considering α = 0.05/8 (adjusted for the number of associations) and 90% power.

### Statistical analysis

The causal relationships were assessed using the IVW method. To enhance this approach, several robust MR methods were used in the sensitivity analysis, including weighted median, weighted mode, and MR Egger methods. The presence of horizontal pleiotropy was evaluated using the Egger intercept method and MR pleiotropy residual sum and outlier (MR-PRESSO).^24^ All analyses were implemented using the “TwoSampleMR” packages in R software. Estimates are detailed in Table 1. All the SNPs used in this study are presented in Table S5.

**Table 1:** Results of the Mendelian randomization analysis of genetically predicted BMD and cardiac diseases.

Cardiovascular outcome	IVW beta	IVW SE	IVW *p*-value	Egger beta	Egger SE	Egger *p*-value	Wme beta	Wme SE	Wme *p*-value	Wmo beta	Wmo SE	Wmo *p*-value	MR_pre *p*-value	Egger intercept *p*-value	SNPs
**UK Biobank**															
** Atrial fibrillation and flutter**	0.011	0.03	0.73	−0.017	0.09	.84	−0.014	0.04	.76	−0.03	0.08	.67	1	.72	76
** Angina**	0.04	0.03	0.17	−0.005	0.08	.95	.041	0.04	.38	0.044	0.07	.55	1	.52	76
** Chronic ischemic heart disease**	0.009	0.03	0.74	0.014	0.07	.85	0.023	0.043	.59	0.031	0.06	.64	1	.94	76
** Heart failure**	0.004	0.04	0.91	−0.023	0.11	.84	−0.027	0.07	.69	−0.011	0.11	.91	0.48	.79	68
** Hypertension**	−0.01	0.01	0.44	0.016	0.03	.65	0.001	0.02	.95	0.012	0.03	.70	0.96	.42	76
** Myocardial infarction**	0.02	0.03	0.36	0.014	0.08	.85	0.033	0.041	.41	0.028	0.06	.64	0.85	.85	81
** Non-ischemic cardiomyopathy**	0.1	0.08	0.20	−0.21	0.21	.34	0.004	0.13	.97	−0.046	0.20	.82	0.58	.11	68
**FinnGen**															
** Atrial fibrillation and flutter**	0.02	0.03	0.45	0.03	0.09	.73	0.06	0.03	.07	0.12	0.05	.04	0.96	.94	79
** Angina**	0.01	0.03	0.62	−0.05	0.08	.52	−0.03	0.03	.26	−0.09	0.05	.11	0.19	.37	79
** Chronic ischemic heart disease**	0.005	0.02	0.84	−0.08	0.07	.26	−0.01	0.02	.47	−0.03	0.04	.39	0.18	.19	79
** Heart failure**	−0.04	0.02	0.13	−0.07	0.07	.33	−0.04	0.03	.23	−0.03	0.05	.54	0.87	.64	79
** Hypertension**	0.004	0.02	0.83	0.02	0.06	.70	0.02	0.02	.34	−0.0007	0.03	.98	0.69	.74	79
** Myocardial infarction**	−0.01	0.03	0.61	−0.02	0.10	0.81	−0.50	0.04	0.21	−0.05	0.06	.37	0.57	.95	79
** Non-ischemic cardiomyopathy**	−0.53	0.03	0.17	−0.03	0.10	0.73	−0.01	0.05	0.80	0.03	0.08	.60	0.17	.85	79

For myocardial infarction, the *p*-value of MR_pre represents the distortion test. Results are presented for the UK Biobank (primary analysis) and FinnGen (validation analysis) cohorts.

Abbreviations: IVW, inverse variance weighted; SE, standard error; MR_pre, Mendelian randomization Pleiotropy RESidual Sum and Outlier test (global test); SNP, single nucleotide polymorphism; Wme, Weighted median; Wmo, Weighted mode

## Results

The results of the power calculation indicate that the MR analysis is robustly powered (90%) to detect even small effect sizes (OR < 1.5). Table S4 provides detailed power calculations for each cardiac outcome, including explained variance, sample size and the proportion of cases. We extracted 85 independent SNPs based on genome-wide significance from the BMD GWAS. A detailed list of each variant can be found in Table S5. Due to the absence of some variants in the GWAS for cardiac conditions, the number of variants in the MR analysis for each cardiac condition varied from 68 to 81 in the UK Biobank dataset. In the FinnGen dataset, the number of variants used was 79 for all cardiac conditions (Table 1).

### Causal effect of total body BMD on CVD

#### Atrial fibrillation and flutter

**Figure 2:**
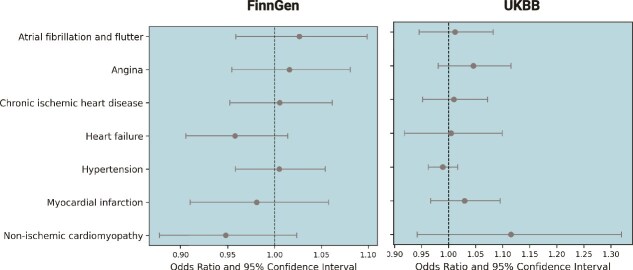
Impact of SNPs on BMD and cardiac diseases. The scatter plots from the Mendelian randomization (MR) analysis illustrate the statistical relationship between genetically predicted BMD and various cardiac diseases. The *x*-axis represents the odds ratio and 95% confidence intervals for the genetic association between BMD and outcomes, derived from both the UK Biobank (UKBB) and FinnGen cohorts. The *y*-axis lists the cardiac outcomes analyzed, including atrial fibrillation and flutter, chronic ischemic heart disease, heart failure, hypertension, myocardial infarction, and non-ischemic cardiomyopathy. The MR methods used include inverse variance weighted, MR-Egger, simple mode, weighted median, and weighted mode. Results are presented separately for each cohort to validate findings across independent datasets.

Table 1 provides the MR estimates from distinct methods assessing the causal effect of genetically predicted BMD on atrial fibrillation and flutter. This analysis incorporated 76 SNPs from the UK Biobank and 79 SNPs from FinnGen as genetic instruments. The findings revealed no significant evidence of a causal effect of genetically predicted BMD on atrial fibrillation and flutter risk, as indicated by the IVW method (beta-estimate: 0.011, SE: 0.03, *p* = .73). Sensitivity analyses corroborated these findings, with the weighted median method yielding a *p*-value of 0.76 and the weighted mode method showing a *p*-value of 0.67. Furthermore, neither the intercept from the MR-Egger analysis (*p* = .72) nor the MR-PRESSO Global test (*p* = 1) indicated the presence of directional horizontal pleiotropy. To validate these findings, we conducted an independent analysis using the FinnGen cohort. Similarly, no evidence for a causal relationship was observed, with the IVW method yielding a beta-estimate of 0.02 (SE: 0.03, *p* = .45). Results from sensitivity analyses, including the weighted median and weighted mode methods, were consistent with the primary analysis and no pleiotropy was detected. Figure 2 provides scatter plots illustrating the estimated effect sizes between BMD and CVDs considered in the study, with results from both the UK Biobank and FinnGen cohorts.

#### Angina

The IVW analysis indicated a potential positive association between genetically predicted BMD and angina, although this was not statistically significant (beta-estimate: 0.04, SE: 0.03, *p* = .17). Similar findings were observed with causal estimates from the weighted median (*p* = .38) and weighted mode (*p* = .55) for genetically predicted BMD on angina. No evidence of directional pleiotropy was detected. The analysis from the FinnGen cohort provided consistent results. The IVW analysis yielded a beta-estimate of 0.01 (SE: 0.03, *p* = .62), demonstrating no significant evidence for a causal effect. Sensitivity analyses, including the weighted median (*p* = .26) and weighted mode (*p* = .11), further supported the null findings. Similar to the UK Biobank, no pleiotropy was detected using the MR-Egger or MR-PRESSO Global test (*p* = .19). Detailed results are presented in Table 1 and illustrated in Figure 2, showing the consistency of estimates between the primary and validation analyses.

#### Chronic ischemic heart disease and myocardial infarction

IVW analysis showed no causal impact on the risk of developing chronic IHD (beta-estimate: 0.009, SE: 0.03, *p* = .74) (Table 1 and Figure 2). Similarly, for myocardial infarction, the IVW analysis also indicated no significant association with genetically predicted BMD (beta-estimate: 0.02, SE: 0.03, *p* = .36). Sensitivity analyses for both conditions supported these findings, with MR-Egger (*p* = .85 for chronic IHD and *p* = .85 for myocardial infarction), weighted median (*p* = .59 and 0.41, respectively) and weighted mode (*p* = .64) showing non-significant results. The analyses included 81 SNPs for myocardial infarction and 76 SNPs for chronic IHD. We conducted further sensitivity analyses after removing the outliers. We found no heterogeneity and horizontal pleiotropy (MR-PRESSO global test *p* = 1 for chronic IHD and MR-PRESSO distortion test *p* = .85 for myocardial infarction). These findings were further validated using the FinnGen cohort, which demonstrated consistent results (Table 1 and Figure 2).

#### Heart failure

The IVW method found no significant association between genetically predicted BMD and heart failure. Similarly, the weighted median and weighted mode regression analyses showed non-significant results (*p* = .69 and *p* = .91, respectively). Furthermore, there was no evidence of directional pleiotropy. In the FinnGen cohort, the IVW analysis indicated no significant causal association (beta-estimate: –0.04, SE: 0.02, *p* = .13). Additional sensitivity analyses, including the weighted median (*p* = .23) and weighted mode (*p* = .54), also yielded non-significant results and no pleiotropy was detected (Table 1 and Figure 1).

#### Hypertension

Genetically predicted BMD was not associated with hypertension (beta-estimate: −0.01, SE: 0.01, *p* = .44) in the IVW analysis. Complementary analyses, including weighted median (*p* = .95) and weighted mode (*p* = .70), also indicated no significant association. No directional pleiotropy was found [MR-Egger regression (*p* = .42) or MR-PRESSO analysis (MR-PRESSO global test *p* = .96)]. These findings were further validated in the FinnGen cohort, with results aligning closely across all methods (Table 1 and Figure 2).

#### Non-ischemic cardiomyopathy

Similarly, the IVW analysis showed no significant association between genetically predicted BMD and NICM (beta-estimate: 0.1, SE = 0.08, *p* = .20) with sensitivity analyses (MR-Egger *p* = .34, weighted median *p* = .97 and weighted mode *p* = .82) reinforcing the null finding. The MR-PRESSO global test (*p* = .58) also indicated no evidence of pleiotropy. Consistent results were obtained across all methods in FinnGen, further supporting the lack of a causal relationship (Table 1 and Figure 2).

## Discussion

### Summary of findings

We present a comprehensive evaluation of the relationship between BMD and cardiovascular health, combining data from several large-scale GWAS. Using summary statistics from a GWASmeta-analysis for BMD and independent cardiovascular outcome datasets from UK Biobank (primary analysis) and FinnGen (validation analysis), we conducted a two-sample MR analysis. Our findings do not support a causal effect of BMD on the risk of AF or flutter, angina, chronic IHD, myocardial infarction, HF, hypertension or NICM. The results suggest that previous associations between BMD and CVD in observational studies may be attributable to unaccounted confounding factors or shared biological mechanisms rather than direct causation.

### Comparison with existing literature

Recent epidemiological and clinical studies showed an association between osteoporosis and CVD. While the association between BMD and angina pectoris has been less explored,^25^ a growing body of research has investigated the link between BMD and myocardial infarction. In a population-based cohort study, Park et al.^26^ showed that lower BMD at various skeletal sites (lumbar spine, femur neck and total hip) was independently associated with a heightened risk of atherosclerotic CVD events, including non-fatal myocardial infarction. This finding is consistent with Wiklund et al.^8^ who reported a modest increase in acute myocardial infarction risk for each standard deviation decrease in BMD. Further strengthening the link, recent MR studies, such as the one conducted by Zhang et al.,^13^ have provided evidence for a causal relationship between CHD, myocardial infarction and BMD. However, in reverse MR analyses, the causal relationship between osteoporosis and CVD was not supported. Our MR study aligns with these findings, demonstrating no evidence of a causal link between BMD, chronic IHD, and angina. These results reinforce the notion that the association observed in previous studies may be attributable to confounding factors or common biological mechanisms rather than a direct causal effect.

Our study provides novel insights into the causal relationship between BMD and AF, addressing an important gap in the literature. Given that both osteoporosis and AF increase in prevalence with age, exploring their potential link is crucial for understanding morbidity in aging populations. For instance, vitamin D deficiency, a known contributor to osteoporosis, has been implicated in cardiovascular morbidity^27^^,^^28^ and is hypothesized to increase the risk of AF through mechanisms such as impaired cardiac autonomic function and the deposition of calcium and phosphate in vascular and valvular cusps.^29^^,^^30^ An observational study by Bhatta et al.^31^ explored this link and found no association between lower forearm BMD and increased AF risk. However, it is important to acknowledge the inherent limitations of observational studies, which are often susceptible to confounding factors and the possibility of reverse causality, making it difficult to establish a clear causal link. Our MR analysis leverages genetic variants as IVs, mitigating these biases and allowing a more robust assessment of causality. The findings revealed no direct causal link between BMD and AF, reinforcing the notion that low BMD is unlikely to play a causal role in AF development. Future studies should explore these common pathways and consider other factors influencing both bone health and cardiovascular risk.

Hypertension, a prevalent cardiovascular condition and an independent risk factor for CVDs,^32^ has gained increasing attention in research exploring the intricate relationship between cardiovascular and skeletal health. Its widespread impact on public health underscores the importance of investigating potential associations with bone health, particularly as emerging evidence remains inconsistent. For instance, a study by Tsuda et al.^33^ showed that BMD in the lumbar spine was significantly decreased in female hypertensive subjects compared to normotensive controls. However, subsequent research involving 2738 female participants aged 50 and over, found no significant association between BMD and hypertension.^34^ Despite these results, our MR analysis sought to examine whether there was a causal link between genetically predicted BMD and hypertension. The findings indicated no causality, a conclusion supported by multiple sensitivity analyses. Moreover, our results were validated using the independent FinnGen cohort, which also showed consistent null results. Several biological pathways may explain the association between BMD and hypertension. In particular, low BMD often correlates with 25 OHD deficiency an independent risk factor for high blood pressure.^35^ Previous research has shown a correlation between low BMD and various metabolic risk factors, such as secondary hyperparathyroidism, oxidative stress, inhibition of vitamin K-dependent matrix proteins, osteopontin, and angiotensin II.^36–38^ These factors may play a role in developing arterial stiffness, hypertension, and osteoporosis. Therefore, while no direct causality exists, understanding these common pathways remains critical for managing the health of individuals at risk of both osteoporosis and CVD.

Finally, our study investigated the causality between genetically predicted BMD and NICM, a phenotype increasingly recognized as a significant cause of heart failure.^39^ Previous studies have suggested that cardiomyopathy may be influenced by metabolic and inflammatory pathways, both of which are also implicated in bone health.^40^ In a previous observational analysis, we identified a potential association between higher estimated BMD (measured via quantitative ultrasound) and lower odds of NICM risk. However, in the current study, our genetic analysis did not support a causal relationship between genetically predicted BMD and NICM.

In summary, our MR analysis adds to the growing body of evidence suggesting that BMD is not causally linked to CVDs. The genetic approach provides a robust framework for exploring these relationships, minimizing the limitations of observational studies and offering a clearer understanding of the interplay between skeletal and cardiovascular health. These findings underscore the need for further research into the mechanisms linking BMD and CVD. Uncovering these mechanisms holds the potential to refine risk stratification strategies and identify novel therapeutic targets for these interconnected conditions.

### Strengths and limitations

Using summary-level data from large-scale GWAS enhances statistical power and reduces the potential for confounding. By applying multiple complementary analyses (MR-Egger, weighted median, weighted mode, and MR-PRESSO) we ensured the robustness and consistency of our findings. However, several limitations should be acknowledged. First, the data sources we used were confined to individuals of European ancestry, which minimized the risk of population structure bias but restricted the generalizability of our findings to other populations. Second, our analysis is based on case-control GWAS designs, which lack a clear time-to-event framework and limit the ability to establish temporality. Conducting longitudinal time-to-event MR analysis would require individual-level genetic and longitudinal CVD data with detailed follow-up, which were not available for our study. Future research should explore time-to-event MR approaches using IVs to better address temporality and strengthen causal inference.

## Conclusion

In summary, this MR study, based on GWAS summary data, found no evidence of a causal relationship between genetically predicted BMD and a broad range of cardiovascular phenotypes. These findings highlight the need for continued research to explore the underlying mechanisms linking BMD with CVD.

## Supplementary Material

Supplemental_material_ziaf037

## Data Availability

Publicly available GWAS summary statistics were used for the MR analyses. Referenced data include databases from FinnGen (https://r10.finngen.fi), GWAS Atlas (https://atlas.ctglab.nl), and GWAS Catalog (https://www.ebi.ac.uk/gwas/home).
