# Supplementary material for: Bone mineral density and cardiovascular diseases: a two-sample Mendelian randomization study
Source: JBMR Plus. 2025 Mar 3;9(5):ziaf037. doi: 10.1093/jbmrpl/ziaf037 (PMC11972088; doi:10.1093/jbmrpl/ziaf037)
Supplement: Supplemental_material_ziaf037 [file supplemental_material_ziaf037.docx]

**SUPPLEMENTAL MATERIAL**

Table of Contents

[Supplementary Table 1: Summary of GWAS for cardiovascular conditions in the UK Biobank cohort 2](#_Toc189927528)

[Supplementary Table 2: Summary of GWAS for cardiovascular conditions in the FinnGen cohort 3](#_Toc189927529)

[Supplementary Table 3: Disease definitions 4](#_Toc189927530)

[Supplementary Table 4: Power calculations for Mendelian Randomization analysis 5](#_Toc189927531)

[Supplementary Table 5: Single-Nucleotide Polymorphisms (SNPs) included in the study for BMD 6](#_Toc189927532)

# **Supplementary Table 1: Summary of GWAS for cardiovascular conditions in the UK Biobank cohort**

| **Cardiovascular condition** | **Year** | **Population** | **Cases** | **Controls** | **Source** |
| --- | --- | --- | --- | --- | --- |
| Angina | 2019 | UK Biobank | 12,114 | 373,585 | Watanabe et al.^17^ |
| Hypertension | 2019 | UK Biobank | 99,665 | 189,642 |  |
| Chronic ischaemic heart disease | 2019 | UK Biobank | 14,456 | 286,335 |  |
| Atrial fibrillation and flutter | 2019 | UK Biobank | 10,986 | 233,904 |  |
| Heart failure | 2019 | UK Biobank | 6,504 | 387,652 | Aragam et al.^18^ |
| Non-ischaemic cardiomyopathy | 2019 | UK Biobank | 1,816 | 388,326 |  |
| Myocardial infarction | 2021 | UK Biobank | 17,505 | 454,212 | Hartiala et al.^19^ |

**Footnote:** The table summarises the GWAS used for cardiovascular conditions in the MR analysis. Abbreviations: MR = Mendelian Randomization; GWAS = genome wide association studies.

## **Supplementary Table 2: Summary of GWAS for cardiovascular conditions in the FinnGen cohort**

| **Cardiovascular condition** | **Cohort** | **Cases** | **Controls** | **Source** |
| --- | --- | --- | --- | --- |
| Angina | FinnGen | 36,875 | 343,079 | Kurki et al.^22^ |
| Hypertension |  | 122,996 | 289,117 |  |
| Chronic ischaemic heart disease |  | 69,008 | 343,173 |  |
| Atrial fibrillation and flutter |  | 50,743 | 210,652 |  |
| Heart failure |  | 29,218 | 381,838 |  |
| Non-ischaemic cardiomyopathy |  | 10,839 | 332,334 |  |
| Myocardial infarction |  | 26,060 | 343,079 |  |

### **Supplementary Table 3: Disease definitions**

| **Source** | **UKB Field ICD10 Code** | **Description** |
| --- | --- | --- |
| **Angina** | | |
| ICD10 | I20 | Angina pectoris |
| **Hypertension** | | |
| ICD10 | I10 | Essential (primary) hypertension |
|  | I11.0 | Hypertensive heart disease with (congestive) heart failure |
|  | I11.9 | Hypertensive heart disease without (congestive) heart failure |
|  | I12.0 | Hypertensive renal disease with renal failure |
|  | I12.9 | Hypertensive renal disease without renal failure |
|  | I13.0 | Hypertensive heart and renal disease with (congestive) heart failure |
|  | I10 | Essential (primary) hypertension |
|  | I13.1 | Hypertensive heart and renal disease with renal failure |
|  | I13.2 | Hypertensive heart and renal disease with both (congestive) heart |
|  | I13.9 | Hypertensive heart and renal disease, unspecified |
|  | I15.0 | Renovascular hypertension |
|  | I15.1 | Hypertension secondary to other renal disorders |
|  | I15.2 | Hypertension secondary to endocrine disorders |
|  | I15.8 | Other secondary hypertension |
| **Ischemic heart disease** | | |
| ICD10 | I24 | Other acute ischaemic heart diseases |
|  | I25 | Chronic ischaemic heart disease |
| **Atrial fibrillation/flutter** | | |
| ICD10 | I48.0 | Paroxysmal atrial fibrillation |
|  | I48.1 | Persistent atrial fibrillation |
|  | I48.2 | Chronic atrial fibrillation |
|  | I48.9 | Atrial fibrillation and atrial flutter, unspecified |
| **Heart failure** | | |
| ICD10 | I50.0 | Congestive heart failure |
|  | I50.1 | Left ventricular failure |
|  | I50.9 | Heart failure, unspecified |
| **Non-ischemic cardiomyopathy** | | |
| ICD10 | I42 | Cardiomyopathy |
|  | I43 | Cardiomyopathy in diseases classified elsewhere |
|  | I11 | Hypertensive heart disease |
|  | I13 | Hypertensive heart and renal disease |
| **Myocardial infarction** | | |
| ICD10 | I21 | Acute myocardial infarction |
|  | I22 | Subsequent myocardial infarction |
|  | I23 | Certain current complications following acute myocardial infarction |
|  | I24 | Other acute ischaemic heart diseases |

**Supplementary Table 2.** ICD: international classification of disease

#### **Supplementary Table 4: Power calculations for Mendelian Randomization analysis**

| **Exposure** | **Explained**  **variance** | **Cardiac outcome** | **Cases** | **Control** | **Sample**  **size** | **Proportion**  **of cases** | **Alpha** | **Power** | **OR** |
| --- | --- | --- | --- | --- | --- | --- | --- | --- | --- |
| BMD | 0.1 | Angina | 12114 | 373585 | 385699 | 0.031407911 | 0.0063 | 0.90 | 1.117 |
|  |  | Hypertension | 99665 | 189642 | 289307 | 0.34449564 |  |  | 1.0509 |
|  |  | Chronic ischemic  heart disease | 14456 | 286335 | 300791 | 0.048059949 |  |  | 1.109 |
|  |  | Atrial fibrillation  and flutter | 10986 | 233904 | 244890 | 0.044860958 |  |  | 1.125 |
|  |  | Heart failure | 6504 | 387652 | 394156 | 0.016501081 |  |  | 1.159 |
|  |  | Nonischemic  cardiomyopathy | 1816 | 388326 | 390142 | 0.004654715 |  |  | 1.298 |
|  |  | Coronary artery  disease | 3968 | 11698 | 15666 | 0.253287374 |  |  | 1.245 |
|  |  | Myocardial infarction | 17505 | 454212 | 471717 | 0.037109114 |  |  | 1.098 |

**Footnote:** This table presents the power calculations for detecting associations between BMD and various cardiac outcomes using MR analysis. The table includes the explained variance of the exposure (BMD), the number of cases and controls, the sample size, the proportion of cases in the sample, the alpha level, power of the study and the OR detectable with 90% power for each cardiac outcome. Abbreviations: BMD = bone mineral density; OR = odds ratio.

##### **Supplementary Table *5*: Single-Nucleotide Polymorphisms (SNPs) included in the study for BMD**

| **Chr** | **Pos** | **rsid** | **EA** | **OA** | **EAF** | **beta** | **pval** | **N** | **StdErr** |
| --- | --- | --- | --- | --- | --- | --- | --- | --- | --- |
| 1 | 8422676 | rs2252865 | t | c | 0.3241 | -0.0328 | 4.72E-08 | 66075 | 0.006 |
| 1 | 22486029 | rs56104760 | a | g | 0.8095 | 0.0747 | 7.38E-24 | 66381 | 0.0074 |
| 1 | 22703035 | rs10493013 | t | c | 0.8189 | -0.1013 | 4.08E-43 | 66572 | 0.0074 |
| 1 | 68656697 | rs2566752 | t | c | 0.6103 | -0.0721 | 1.88E-34 | 66398 | 0.0059 |
| 1 | 68664913 | rs2566751 | a | t | 0.8727 | -0.0567 | 1.32E-08 | 66398 | 0.01 |
| 1 | 110475971 | rs7548588 | t | c | 0.609 | -0.0367 | 2.21E-10 | 66240.1 | 0.0058 |
| 1 | 172186729 | rs633995 | a | g | 0.4251 | 0.0351 | 1.61E-09 | 66119 | 0.0058 |
| 1 | 219897941 | rs4846580 | a | g | 0.5329 | 0.0345 | 3.21E-09 | 66614 | 0.0058 |
| 1 | 240581653 | rs12044944 | t | c | 0.1916 | 0.0553 | 7.54E-14 | 65125 | 0.0074 |
| 2 | 27741072 | rs780096 | c | g | 0.4406 | -0.0311 | 4.58E-08 | 66578 | 0.0057 |
| 2 | 40630678 | rs10490046 | a | c | 0.7609 | 0.0429 | 1.43E-10 | 65961 | 0.0067 |
| 2 | 42284110 | rs2289410 | a | t | 0.868 | 0.0494 | 2.00E-08 | 66494 | 0.0088 |
| 2 | 54684557 | rs11898505 | a | g | 0.3326 | 0.0342 | 1.28E-08 | 66091 | 0.006 |
| 2 | 68962137 | rs10048745 | a | g | 0.2477 | -0.0389 | 6.44E-09 | 66565.1 | 0.0067 |
| 2 | 85484818 | rs11904127 | a | g | 0.5512 | -0.0324 | 1.18E-08 | 66561 | 0.0057 |
| 2 | 119548256 | rs144279715 | a | g | 0.9853 | -0.2295 | 6.18E-15 | 64027 | 0.0294 |
| 2 | 119632252 | rs12612325 | a | g | 0.2122 | -0.0548 | 1.98E-12 | 66509 | 0.0078 |
| 2 | 166577489 | rs7586085 | a | g | 0.5337 | 0.0532 | 8.64E-21 | 66609 | 0.0057 |
| 2 | 202799604 | rs2350085 | t | c | 0.8726 | -0.0643 | 3.80E-14 | 66412 | 0.0085 |
| 2 | 202832130 | rs10931982 | t | c | 0.2097 | -0.0508 | 1.59E-08 | 55344.1 | 0.009 |
| 2 | 234303405 | rs838721 | a | g | 0.437 | -0.0314 | 4.48E-08 | 65515.9 | 0.0057 |
| 3 | 41127046 | rs447911 | c | g | 0.5348 | 0.0708 | 6.29E-36 | 66564 | 0.0057 |
| 3 | 156692207 | rs74394007 | a | c | 0.862 | 0.0608 | 2.46E-13 | 66607 | 0.0083 |
| 4 | 1006987 | rs76051363 | t | c | 0.1491 | -0.0794 | 1.39E-20 | 60802 | 0.0085 |
| 4 | 88831249 | rs11934731 | a | g | 0.6738 | -0.0674 | 8.39E-29 | 66623 | 0.0061 |
| 5 | 88288341 | rs7728694 | t | g | 0.461 | -0.0503 | 1.30E-17 | 66527 | 0.0059 |
| 5 | 112221869 | rs818427 | t | c | 0.3118 | 0.0342 | 2.37E-08 | 66592.9 | 0.0061 |
| 5 | 122847622 | rs11745493 | a | g | 0.7463 | 0.0445 | 7.75E-12 | 66597 | 0.0065 |
| 6 | 44636919 | rs7741085 | t | c | 0.5874 | 0.0423 | 1.51E-13 | 66441.1 | 0.0057 |
| 6 | 127167072 | rs13204965 | a | c | 0.771 | 0.0619 | 1.02E-18 | 66132.9 | 0.007 |
| 6 | 151910126 | rs6557155 | t | g | 0.4318 | -0.0751 | 2.56E-37 | 66602 | 0.0059 |
| 6 | 151971720 | rs7740042 | a | t | 0.2024 | -0.0494 | 2.71E-12 | 66602 | 0.0071 |
| 7 | 27989403 | rs757138 | t | g | 0.6889 | -0.0348 | 3.33E-08 | 66043.1 | 0.0063 |
| 7 | 30997087 | rs73305797 | a | t | 0.7569 | 0.0422 | 2.40E-10 | 66180 | 0.0067 |
| 7 | 38142840 | rs34102936 | a | g | 0.5897 | 0.0471 | 1.87E-16 | 66579 | 0.0057 |
| 7 | 50901491 | rs1548607 | a | g | 0.687 | 0.0363 | 4.18E-08 | 66564 | 0.0066 |
| 7 | 96134115 | rs6465511 | c | g | 0.3248 | -0.0738 | 1.03E-34 | 66612 | 0.006 |
| 7 | 96660132 | rs6960249 | t | g | 0.5909 | 0.0325 | 1.45E-08 | 66292 | 0.0057 |
| 7 | 99130834 | rs34670419 | t | g | 0.0394 | -0.088 | 1.09E-08 | 66336 | 0.0154 |
| 7 | 120730944 | rs12534510 | a | c | 0.4455 | -0.0395 | 3.15E-12 | 66614 | 0.0057 |
| 7 | 120974765 | rs3801387 | a | g | 0.7279 | -0.1347 | 1.15E-100 | 66004 | 0.0063 |
| 7 | 121191251 | rs73719807 | a | c | 0.9129 | -0.0925 | 1.14E-16 | 66582 | 0.0112 |
| 7 | 150953205 | rs73169678 | a | c | 0.1117 | 0.0619 | 1.05E-11 | 66472 | 0.0091 |
| 8 | 120012700 | rs11995824 | c | g | 0.4319 | 0.0675 | 1.06E-31 | 66121 | 0.0058 |
| 9 | 54412493 | rs1159798 | a | c | 0.2402 | 0.0429 | 1.01E-09 | 60898 | 0.007 |
| 9 | 133471891 | rs10901216 | a | g | 0.3438 | -0.0474 | 5.53E-15 | 66354 | 0.0061 |
| 10 | 54423853 | rs12258451 | c | g | 0.8693 | 0.0702 | 2.41E-15 | 66519 | 0.0089 |
| 10 | 112245400 | rs73349318 | a | t | 0.8738 | -0.0472 | 2.68E-08 | 66341 | 0.0085 |
| 10 | 124015986 | rs10788264 | a | g | 0.4815 | -0.0338 | 2.61E-09 | 66565 | 0.0057 |
| 11 | 242859 | rs55781332 | a | g | 0.7831 | -0.0552 | 8.07E-16 | 66198 | 0.0069 |
| 11 | 15708792 | rs7926837 | a | g | 0.7854 | -0.0564 | 3.98E-16 | 66568 | 0.0069 |
| 11 | 15816918 | rs10832520 | a | t | 0.0394 | 0.1123 | 1.00E-12 | 66628 | 0.0158 |
| 11 | 16630779 | rs35199438 | t | g | 0.3035 | -0.0489 | 2.36E-15 | 66609.9 | 0.0062 |
| 11 | 27306364 | rs7105860 | c | g | 0.6025 | -0.0468 | 2.36E-15 | 66376 | 0.0059 |
| 11 | 35083633 | rs2553773 | c | g | 0.4137 | -0.037 | 1.49E-10 | 66619 | 0.0058 |
| 11 | 46766890 | rs61884327 | t | c | 0.9022 | -0.0801 | 4.64E-16 | 66175 | 0.0099 |
| 11 | 47284279 | rs143187557 | t | c | 0.0223 | -0.1237 | 1.15E-09 | 66321 | 0.0203 |
| 11 | 68218290 | rs11228240 | t | c | 0.2574 | -0.083 | 1.72E-35 | 66583 | 0.0067 |
| 11 | 86887931 | rs634277 | a | g | 0.6678 | 0.0607 | 2.15E-23 | 66585 | 0.0061 |
| 11 | 121913230 | rs725670 | a | g | 0.383 | -0.0322 | 3.61E-08 | 66565.9 | 0.0059 |
| 12 | 1639249 | rs35125553 | a | g | 0.7145 | -0.0383 | 5.20E-09 | 66278 | 0.0066 |
| 12 | 49379537 | rs118115924 | t | g | 0.0139 | -0.2822 | 6.99E-21 | 58918 | 0.0301 |
| 12 | 49655948 | rs117557198 | a | g | 0.9324 | -0.0769 | 1.58E-10 | 66523 | 0.012 |
| 12 | 53743064 | rs10735851 | a | g | 0.7083 | -0.0541 | 5.84E-18 | 66566 | 0.0063 |
| 12 | 90334829 | rs10777212 | t | g | 0.3455 | 0.0452 | 5.05E-14 | 66619 | 0.006 |
| 12 | 107302778 | rs1037011 | t | c | 0.4792 | -0.0404 | 1.54E-12 | 66616 | 0.0057 |
| 13 | 42952145 | rs9594738 | t | c | 0.4592 | -0.0614 | 3.84E-27 | 66157 | 0.0057 |
| 13 | 43200103 | rs78667121 | a | g | 0.0325 | 0.1326 | 1.70E-13 | 66346 | 0.018 |
| 14 | 91464890 | rs1286150 | t | c | 0.8045 | -0.0549 | 2.44E-14 | 66573 | 0.0072 |
| 15 | 38340874 | rs12442242 | a | g | 0.8501 | -0.0509 | 4.94E-10 | 66418 | 0.0082 |
| 15 | 51537806 | rs2414098 | t | c | 0.3898 | -0.0329 | 1.99E-08 | 66562.1 | 0.0059 |
| 15 | 67547301 | rs3743347 | a | c | 0.2351 | 0.0519 | 1.75E-14 | 66615.1 | 0.0068 |
| 16 | 392318 | rs8047501 | a | g | 0.4923 | 0.0524 | 1.13E-18 | 66340 | 0.0059 |
| 16 | 86714715 | rs71390846 | c | g | 0.1836 | -0.0484 | 1.38E-10 | 65285 | 0.0075 |
| 17 | 2064702 | rs2873195 | a | t | 0.3127 | -0.0406 | 4.31E-11 | 66572 | 0.0062 |
| 17 | 17804725 | rs8070128 | t | c | 0.5763 | -0.0394 | 1.98E-11 | 66625 | 0.0059 |
| 17 | 41819562 | rs144691710 | a | g | 0.9249 | -0.1017 | 2.24E-19 | 66392 | 0.0113 |
| 17 | 42283037 | rs9910055 | t | c | 0.2624 | 0.0442 | 3.12E-11 | 66576.1 | 0.0067 |
| 17 | 60054857 | rs884205 | a | c | 0.2421 | -0.0531 | 4.39E-15 | 66040 | 0.0068 |
| 17 | 63771079 | rs9972944 | a | g | 0.4049 | 0.0363 | 6.87E-10 | 66595 | 0.0059 |
| 20 | 10640877 | rs6040063 | a | g | 0.5007 | 0.0359 | 1.78E-10 | 66499 | 0.0056 |
| 20 | 39103882 | rs6029130 | t | c | 0.2874 | 0.0348 | 3.50E-08 | 66497 | 0.0063 |
| 21 | 28773868 | rs1452102 | t | g | 0.5871 | -0.0345 | 1.74E-09 | 66489 | 0.0057 |
| 21 | 36970350 | rs9976876 | t | g | 0.447 | -0.0375 | 8.01E-11 | 66514 | 0.0058 |
| 21 | 40350744 | rs11910328 | a | g | 0.8351 | -0.0429 | 2.99E-08 | 66298 | 0.0077 |

**Footnote:** Chr: Chromosome; Pos: position; rsid: variant ID; EA: effect allele; OA: other allele; EAF: effect allele frequency; pval: P-value; StdErr: standard error; N: sample size.
